# Smoking in cars in England: a study of school students in an English city

**DOI:** 10.1186/1471-2458-14-559

**Published:** 2014-06-05

**Authors:** Ilze Bogdanovica, Lisa Szatkowski, John Britton, Ann McNeill

**Affiliations:** 1 UK Centre for Tobacco and Alcohol Studies, University of Nottingham, Nottingham, NG5 1 PB, United Kingdom; 2 Division of Epidemiology and Public Health, Clinical Sciences Building, City Hospital, Nottingham, NG5 1 PB, United Kingdom; 3 UK Centre for Tobacco and Alcohol Studies/Addictions Department, Institute of Psychiatry. King’s College London, 4 Windsor Walk, Denmark Hill, London SE5 8BB, United Kingdom

**Keywords:** Smoking in cars, England, Deprivation

## Abstract

**Background:**

Exposure to secondhand smoke is associated with an increased risk of adverse health effects among children. Although smoking in the home is an established major source of exposure, less is known about rules on smoking in cars.

**Methods:**

In a survey including a sample of secondary school students in Nottingham (UK) in 2012, participants were asked whether smoking was allowed in the family car, and how often the respondent travelled in a car in which smoking was allowed. Rules on smoking in cars were investigated in relation to socio-demographic variables and whether children had ever smoked themselves using logistic regression.

**Results:**

Of 4,190 students aged 11–16 who provided data, approximately 12% reported that smoking was allowed in their family car and 35% that they travelled in a car where smoking was allowed at least sometimes. Absence of smoke free rules in the family car was more likely to be reported by children from more disadvantaged families, if parents and friends were smokers and if smoking was allowed in the main home. These factors, and having a sibling who smokes, were also independently associated with an increased risk of travelling in a car in which smoking was allowed at least sometimes. Respondents who were not protected from secondhand smoke in the car were also more likely to have ever smoked (adjusted odds ratio 1.59, 95% CI 1.18-2.14).

**Conclusions:**

Absence of smoke free rules in a family car and travelling in a car where smoking was allowed was relatively common among secondary school students, was strongly related to social disadvantage and a higher risk of smoking experimentation. Measures to prevent such exposure are therefore indicated.

## Background

Smoking is a major cause of preventable death in England, leading to more than 80,000 deaths a year (2009 data) [1]. Along with the direct harmful effects of smoking, exposure to secondhand smoke (SHS) is a significant cause of morbidity and mortality both among adults and children. In children, exposure to secondhand smoke is associated with an increased risk of sudden infant death, lower respiratory tract infections, middle-ear disease, and exacerbation of asthma [2]. It has been reported that the risk of adverse health effects for children increases with the number of smokers in the household [2], and that exposure occurs in particular in the home and the family car [3]. It has also been reported that for children exposed to smoking in cars there is a higher risk of developing respiratory symptoms [4].

Although smoking restrictions in various public places have been introduced, smoking while driving is still common and often occurs in the presence of adult non-smokers and children. Typically, smoke free rules in a car are more common among non-smokers than smokers [5], for example, in the Netherlands 36% of smokers allow smoking in cars carrying children [6]. In the UK, nearly a third of smokers smoke in their car when non-smokers are present [7]. However, in some countries, including Australia and some parts of Canada, legislation has been implemented to prohibit smoking in cars when children are present to protect them from exposure to tobacco smoke. Although in the UK smoking is restricted in vehicles used for work, smoking in private cars is not restricted even when children are present [8].

Although findings from earlier studies suggest that a considerable proportion of smokers smoke in their cars, there is limited up to date evidence on the extent to which children are protected from SHS in cars in England and what factors are associated with smoke free rules in cars. This study has therefore estimated the proportion of children from a large city in England travelling in cars where smoking is allowed, and identified factors associated with smoking being allowed in family car and frequency of travelling in cars where smoking is allowed, and with ever smoking among participants.

## Methods

### Data collection

Data were collected as part of a second wave of a cohort survey (first study described elsewhere [9]) carried out in March 2012 in Nottingham (UK). The initial sample of 11 schools surveyed in 2011 was based on a convenience sample (11 of all 36 schools initially contacted in the area agreed to participate). We invited all 11 schools to participate in 2012 survey, and eight schools agreed to do so. In these schools, information sheets were distributed to head teachers and parents of children in school years 7–11 (aged 11–16). Students (more than 6000) were then invited to participate in the study unless they or their parents denied consent. Children were asked to fill in the questionnaire during their usual school activities. Some schools did not survey all classes, but we do not have information on this or children who did not take part in the study because they were absent. In total we received 4,302 completed questionnaires, and the response rate was approximately 69%.

Two questions on smoking in cars were asked: “Is smoking allowed in your family car?” (with response categories yes/no/my family doesn’t have a car) and “How often do you travel in a car where smoking is allowed?” (with response categories every day/most days/some days/on the odd day/never). The questionnaire also contained questions to gather data on demographic characteristics, school year, smoking (ever vs. never smoking), deprivation (measured as Index for Multiple Deprivation (IMD [10]), which includes weighted estimates for income deprivation, employment deprivation, health deprivation and disability, education deprivation, barriers to housing and services, crime and living environment [11]), smoking rules in the home (allowed vs. not allowed), and smoking among family members (neither of parents, one parent or both parents; at least one vs. none for siblings) and friends (none, one or two, three or more, not sure).

### Statistical analysis

Univariable logistic regression models were built to examine the association between each explanatory variable (sex, school year, deprivation, parental smoking, sibling smoking, smoking rules in the home and number of smoking friends) and each of the two questions on smoking in cars. For the question on whether smoking is allowed in the family car the outcome measure was dichotomised as allowed vs. not allowed, and for the question on frequency of travelling in a car where smoking is allowed the outcome was dichotomized as travelling at least sometimes vs. never. We accounted for the non-independence and possibly more similar characteristics of students clustered within a school compared to those in other schools by calculating robust confidence intervals around our odds ratios using the clustered sandwich estimator [12,13]. Variables that were significant at the univariable level were considered for inclusion in a multivariable logistic regression model. Likelihood ratio tests were used to determine which of these variables should be included in the final multivariable model. The level of statistical significance was set at p < 0.05, and 95% confidence intervals were calculated.

For the analysis investigating whether smoking was allowed in the family car, those who reported that their family does not have a car or did not respond to this question were excluded from the analysis. When frequency of travelling in a car where smoking is allowed was investigated, participants who did not respond to this question were excluded from the analysis. In order to ensure that the sample size for the analysis was as large as possible missing values for explanatory variables were coded as a separate category and included in the analysis. A complete case analysis was carried out as a sensitivity analysis.

We also used logistic regression to examine the association between car smoking rules and travelling in a car where smoking is allowed and children’s smoking status (ever vs. never smoking), adjusting for other variables as above. Here, missing values for the smoking in car questions were coded as a separate category and included in the analysis but missing values for the outcome variable, ever smoking, were excluded.

Data were analysed using Stata v.11 (Stata Corp. College Station, TX). Ethics approval for this study was granted by the University of Nottingham School of Education Research Ethics Committee.

## Results

Out of 4,302 questionnaires received, 205 were from students who reported that their family does not have a car; 79 provided no data on either of the two questions on smoking in cars; 33 did not answer the question on whether smoking is allowed in their family car; and 31 did not report how often they travel in a car where smoking is allowed. Our findings suggest that there was a statistically significant difference in the frequency of traveling in a car where smoking was allowed in relation to smoking rules in a family car (Chi square 936.94, p < 0.001). While 73.6% of children from families where smoking in a car was not permitted never travelled in a car where smoking was allowed, only 4.1% of children with no rules banning smoking in a family car never travelled in a car where smoking was allowed and the majority of them (95.9%) at least sometimes travelled in a car where smoking was allowed. There were almost equal proportions of boys and girls in our sample including children from school years 7–11 (aged 11–16). For the majority of children, family members (parents, siblings) were non-smokers and smoking was not allowed in the family home. However, for more than 40% of children at least one of their friends was a smoker (Table 1 & Table 2).

**Table 1:** Unadjusted and adjusted odds of smoking being allowed in the family car (excluding children whose family does not have a car)

**Variable**	**Number of students (% where smoking allowed)**	**Univariable model**			Multivariable model*		
		**Odds ratio**	**Confidence interval**	**P-value**	**Odds ratio**	**Confidence interval**	**P-value**
**Sex**							
Boy	1,964 (12.1)	1.00		0.287			
Girl	2,003 (13.7)	1.15	1.02-1.31				
Missing	18 (16.7)	1.45	0.34-6.16				
**School year**							
Year 7	589 (12.2)	1.00		0.063			
Year 8	1,021 (13.9)	1.16	0.91-1.48				
Year 9	984 (10.8)	0.87	0.61-1.24				
Year 10	972 (13.8)	1.15	0.81-1.63				
Year 11	408 (14.2)	1.19	0.82-1.73				
Missing	11 (36.4)	4.10	0.84-20.0				
**Deprivation**							
V (least deprived)	1,026 (4.9)	1.00		<0.001	1.00		<0.001
IV	437 (10.8)	2.35	1.72-3.21		1.85	1.11-3.06	
III	534 (12.2)	2.70	1.72-4.26		1.52	0.95-2.43	
II	467 (15.2)	3.50	2.56-4.79		2.29	1.43-3.66	
I (most deprived)	444 (22.1)	5.53	2.58-11.86		2.51	1.60-3.94	
Missing	1,077 (17.2)	4.05	2.61-6.27		2.17	1.45-3.23	
**Parental smoking**							
Neither parent smokes	2,771 (3.1)	1.00		<0.001	1.00		<0.001
One parent smokes	841 (30.0)	13.20	9.07-19.20		6.08	4.56-8.11	
Both parents smoke	346 (50.6)	31.57	21.77-45.78		9.83	6.95-13.90	
Missing	27 (7.4)	2.47	0.82-7.43		1.75	0.37-8.34	
**Sibling smoking**							
None smokes	3,511 (10.6)	1.00		<0.001			
At least one smokes	447 (32.0)	3.98	3.12-5.08				
Missing	27 (7.4)	0.68	0.19-2.35				
**Smoking in the main home**							
Not allowed	3,376 (5.0)	1.00		<0.001	1.00		<0.001
Allowed	583 (58.3)	26.39	20.04-34.74		11.03	8.59-14.18	
Missing	26 (23.1)	5.66	3.03-10.57		3.48	1.26-9.62	
**Friend smoking**							
None	1,350 (6.4)	1.00		<0.001	1.00		<0.001
One or two	558 (13.3)	2.25	1.76-2.86		1.51	1.00-2.28	
Three or more	1,114 (22.0)	4.14	3.47-4.94		2.20	1. 59–3.05	
Not sure	940 (11.5)	1.91	1.48-2.47		1.44	1.00-2.07	
Missing	23 (13.0)	2.20	0.46-10.53		1.41	0.31-6.35	

*Only variables significant at multivariable level are presented for the multivariable model in this table.

**Table 2:** Unadjusted and adjusted odds of travelling in a car where smoking is allowed*

**Variable**	**Number of students (% travel in car where smoking allowed)**	**Univariable model**			**Multivariable model**		
		**Odds ratio**	**Confidence interval**	**P-value**	**Odds ratio**	**Confidence interval**	**P-value**
**Sex**							
Boy	2,072 (33.6)	1.00		0.044			
Girl	2,100 (37.3)	1.17	1.02-1.35				
Missing	20 (40.0)	1.32	0.67-2.59				
**School year**							
Year 7	623 (31.0)	1.00		0.016			
Year 8	1,078 (35.4)	1.22	0.91-1.65				
Year 9	1,023 (34.9)	1.19	0.87-1.63				
Year 10	1,027 (36.1)	1.26	0.98-1.61				
Year 11	430 (41.9)	1.60	1.10-2.34				
Missing	11 (45.6)	1.86	0.33-10.47				
**Deprivation**							
V (least deprived)	1,040 (26.6)	1.00		<0.001	1.00		0.009
IV	446 (33.2)	1.37	1.05-1.78		1.13	0.87-1.46	
III	550 (37.5)	1.65	1.24-2.20		1.23	0.96-1.57	
II	501 (36.9)	1.61	1.30-2.00		1.15	0.90-1.48	
I (most deprived)	507 (48.5)	2.60	1.55-4.36		1.58	1.23-2.03	
Missing	1,148 (37.1)	1.63	1.19-2.21		1.05	0.86-1.27	
**Parental smoking**							
Neither parent smokes	2,856 (24.4)	1.00		<0.001	1.00		<0.001
One parent smokes	911 (55.4)	3.85	3.31-4.48		2.41	2.03-2.87	
Both parents smoke	395 (70.9)	7.54	5.92-9.61		3.37	2.60-4.38	
Missing	30 (20.0)	0.77	0.35-1.70		0.60	0.24-1.51	
**Sibling smoking**							
None smokes	3,658 (32.7)	1.00		<0.001	1.00		0.006
At least one smokes	504 (56.9)	2.73	2.09-3.55		1.36	1.10-1.70	
Missing	30 (20.0)	0.52	0.25-1.06		Omitted due to collinearity		
**Smoking in the main home**							
Not allowed	3,495 (27.9)	1.00		<0.001	1.00		<0.001
Allowed	660 (74.9)	7.70	6.99-8.49		3.89	3.14-4.81	
Missing	37 (54.1)	3.05	1.90-4.88		2.32	1.16-4.66	
**Friend smoking**							
None	1,390 (24.0)	1.00		<0.001	1.00		<0.001
One or two	594 (39.4)	2.06	1.72-2.47		1.67	1.33-2.09	
Three or more	1,179 (48.9)	3.03	2.43-3.78		2.09	1.73-2.51	
Not sure	1,004 (33.7)	1.61	1.32-1.96		1.34	1.10-1.62	
Missing	25 (28.0)	1.23	0.60-2.52		0.93	0.36-2.44	

*Only variables significant at multivariable level are presented for the multivariable model in this table.

### Smoking rules in the family car

Of 3,985 students with a family car who responded to the question on whether smoking was allowed in the family car, 12.9% (95% CI 7.8-18.1) reported that smoking was allowed in it. In univariable analysis there was a strong association between the likelihood of smoking being allowed in the family car and increasing deprivation, the number of parents who were smokers, having siblings or friends who smoke, and in families where smoking was allowed in the main home (Table 1). In a mutually adjusted multivariable regression, significant predictors of smoking being allowed in the family car were deprivation, parental smoking, smoking rules in the main home and friends smoking (Table 1).

### Travelling in a car where smoking is allowed

Although the majority (64.5%; n = 2,704) of students who answered the question on the frequency of travelling in a car where smoking was allowed (n = 4,192) reported that they never travel in a car where smoking was allowed, 35.4% (95% CI 29.1-41.9) reported that they travel in a car where smoking was allowed at least sometimes. This proportion included 2.6% who travel in a car where smoking was permitted every day, 3.9% on most days, 7.9% on some days and 21.1% on the odd day. Factors associated with the frequency of travelling in a car where smoking was allowed at the univariable level included being a girl, being in a higher school year, being from a more deprived background, having smoking parents and siblings, smoking being allowed in the family home and having a greater number of smoking friends (Table 2). In the multivariable analysis, travelling in a car in which smoking was allowed was independently associated with deprivation, parental smoking, sibling smoking, smoking in the main home and friends smoking (Table 2).

### The association between smoking in cars and smoking status

Data on ever smoking were available for 4,170 survey participants, and those who did not report their smoking behaviour were excluded from further analysis. Although the majority of participants were never smokers, 20.9% of all respondents reported that they were ever smokers. Smoking being allowed in a family car was a significant predictor of being an ever smoker (OR = 1.59; 95% CI 1.18-2.14) after adjustment for a range of explanatory factors (sex, school year, deprivation, parental smoking, sibling smoking, smoking rules at home and friends smoking). Also, travelling in a car where smoking was allowed at least sometimes was a significant predictor of being an ever smoker (OR = 1.39; 95% CI 1.12-1.73) after adjusting for the variables mentioned above and whether smoking was allowed in the family car.

For all findings reported, sensitivity analyses using complete cases only were carried out; however these did not reveal considerably different results.

## Discussion and conclusions

The results from this study of a sample of English schoolchildren demonstrate that absence of smoke- free rules in family cars or other cars carrying children is still relatively common, and strongly related to social disadvantage. To our knowledge this is the first large study in England in recent years investigating rules regarding smoking in family and other cars in relation to a range of socio-economic and demographic variables, and smoking in their immediate social environment. Considering the effects of passive smoking on children’s health [3], smoking rules in family cars and potential exposure to secondhand smoke is an important public health issue.

Our study had some limitations. Due to the nature of the questions asked our measures of smoking in cars do not necessarily mean that in all cases respondents are exposed to tobacco smoke. However it is likely that exposure and frequency of travelling in a car where smoking is allowed and rules in the family car will be related. Also, although we had a sample of schools that are likely to be representative to Nottinghamshire, generalizability of the results to the rest of England and other parts of the United Kingdom might be questioned.

According to the national survey in 2011 25% of children aged 11–15 in England were ever smokers [14] while in our study, prevalence of ever smoking among children aged 11–16 was lower, at 21%. Similarly, our figures suggesting that nearly 15% of children were travelling in a car where smoking was allowed at least on some days are consistent with a finding from the national survey that 19% were often near people smoking in cars [14] and findings from the UK Youth Tobacco Policy Survey suggesting that 17% of 11–16 year old adolescent are exposed to smoking in cars more than once a week [15].

Although the findings from our analyses of associations between smoking in car rules and other determinants are of limited causal inference as a result of the cross-sectional study design, the associations with family and sibling smoking, deprivation [16] and lack of smoking rules in the home [17], indicate that smoking being allowed in cars was most common among children from families in which smoking is the norm. Recent estimates from 2012 national survey in England suggest that children from more deprived backgrounds are more likely to be exposed to smoking in cars [18]. The substantial discrepancies observed between the proportion of children with smoking allowed in the family car, and the proportion travelling in a car where smoking is allowed at least sometimes, highlight the importance of SHS in the cars of friends or relatives as a source of exposure in children, and therefore that preventing exposure will require policies covering all cars. It is also apparent from our findings that travelling in cars where smoking is allowed at least sometimes, and having no restrictions on smoking in the family car, were both associated with ever having smoked. Although based on cross-sectional data, these findings are consistent with previously reported evidence that exposure to smoking in cars is related to current smoking and smoking initiation among adolescents [19], and provide further support for comprehensive policies to prevent all forms of exposure.

Our findings are also in line with observation from other studies. In a repeated cross sectional study in New Zealand a slightly greater proportion of children (23%) have been reported to be exposed to smoking in cars in the previous week, however over time a slow decline of the proportion exposed has been observed [20]. Similarly, decline in exposure to smoking in cars over time has been observed among children in the United States [21,22]. In New Zealand greater exposure to secondhand smoke was observed among children from lower socio-economic groups, and smoking being common in family and among friends, and being exposed to smoking at home or in a car was related to increased susceptibility to smoking and risk of becoming a smoker [20].

Apart from the serious health effects caused by smoking in the car on both smokers and non-smokers, one study has shown that smoking while driving is also associated with safety issues as smoking in the car distracts the driver, which leads to an increased risk of motor vehicle accidents among smokers [23]. This, together with the high level of public support for smoke-free car policies, including among children [15,24,25], and our results indicating the high prevalence of children unprotected by smoke-free car rules, particularly among more disadvantaged children and the potential influence on smoking uptake suggests that smoking in cars should be restricted.

## Abbreviations

SHS: Secondhand smoke; OR: Odds ratio; CI: Confidence interval; UK: United Kingdom.

## Competing interests

The authors declare that they have no competing interests.

## Authors’ contributions

IB was involved in designing the study, collecting and analysing the data, and prepared first draft of the manuscript. LS participated in designing statistical analysis of the data and contributed to the manuscript. AM and JB were involved in designing the study, collecting the data and helped to draft the manuscript. All authors contributed to the final manuscript and have approved its publication.

## Pre-publication history

The pre-publication history for this paper can be accessed here:

http://www.biomedcentral.com/1471-2458/14/559/prepub
